# Mycogenic synthesis of ZnO nanoparticles using an endophytic Aspergillus niger isolate from Celastrus paniculatus: evaluation of multifunctional bioactivity for agricultural and antimicrobial applications

**DOI:** 10.1186/s11671-026-04670-y

**Published:** 2026-05-24

**Authors:** Sneha Dwivedi, Mukul Machhindra Barwant, Usman Mohammed Ali, Abdela Tufa

**Affiliations:** 1 Department of Botany, University of Allahabad, Prayagraj, India; 2 Department of Botany, Sanjivani Rural Education Society’s, Sanjivani Arts Commerce and Science College Kopargaon Maharshtra, Kopargaon, 423603 India; 3 Faculty of Agriculture, Department of Plant Sciences, Wollega University, Shambu, Oromia Ethiopia

**Keywords:** Antimicrobial activity, Fungal endophyte, Green synthesis, Nanoparticles, Sustainable agriculture, Zinc oxide

## Abstract

**Background:**

The green synthesis of zinc oxide nanoparticles (ZnO NPs) using biological systems offers an eco-friendly alternative to conventional chemical methods. Fungal endophytes, which produce diverse bioactive metabolites, represent promising resources for nanomaterial synthesis. This study investigates the use of an endophytic *Aspergillus niger* strain isolated from the medicinal plant *Celastrus paniculatus* for the mycogenic synthesis of ZnO NPs and evaluates their multifunctional bioactivity.

**Methods:**

ZnO NPs were synthesized via a sol-gel method using an aqueous extract of *A. niger*. Comprehensive characterization was performed using XRD, FTIR, SEM, TEM, UV-Vis, and PL spectroscopy. Biological activities were assessed through DPPH antioxidant assay, antimicrobial testing (disc diffusion and MIC against bacterial pathogens), antifungal assay against *Fusarium oxysporum*, and plant growth promotion studies on *Oryza sativa* L. Basmati seedlings in a hydroponic system.

**Results:**

Characterization confirmed the formation of pure, hexagonal wurtzite ZnO NPs with an average crystallite size of 27.3 ± 3.1 nm and a band gap of 3.26 eV. FTIR indicated the presence of fungal-derived biomolecules on the NP surface. The NPs exhibited antioxidant activity (IC₅₀ = 42.7 ± 1.8 µg/mL), antibacterial effects (with MIC values of 0.41–3.33 µg/mL against tested strains), and antifungal activity (47.7 ± 1.0% inhibition of *F. oxysporum* at 200 µg/mL). In hydroponic studies, treatment with 2 µM ZnO NPs was associated with modest increases in rice seedling fresh weight (*p* = 0.023) and photosynthetic efficiency (Fv/Fm, *p* = 0.017) compared to controls.

**Conclusion:**

This work demonstrates the synthesis of ZnO NPs using an endophytic *A. niger* isolate from *C. paniculatus*. The synthesized NPs exhibit multiple bioactivities in vitro, suggesting potential for further investigation in agricultural and antimicrobial contexts. However, substantial additional research including cytotoxicity assessment, environmental fate studies, and field trials will be necessary before practical applications can be considered.

**Graphical abstract:**

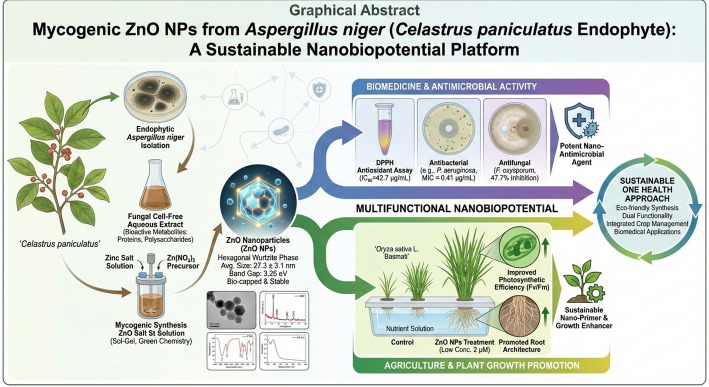

**Supplementary Information:**

The online version contains supplementary material available at 10.1186/s11671-026-04670-y.

## Introduction

Nanotechnology has emerged as a transformative field with implications across medicine, agriculture, and environmental science [1]. Among nano-materials, zinc oxide nanoparticles (ZnO NPs) have attracted attention due to their physicochemical properties, biocompatibility, and potential applications in various sectors [2, 3]. However, conventional synthesis methods often involve toxic chemicals and high energy consumption, raising environmental and safety concerns [4]. This has motivated interest in green synthesis approaches that utilize biological systems such as plants, algae, and microorganisms [5].

Fungi have been investigated as biofactories for nanoparticle synthesis due to their metal tolerance, secretory capabilities, and scalability [6]. Fungal endophytes symbiotic fungi residing within plant tissues are of particular interest as they produce diverse secondary metabolites (e.g., flavonoids, phenols, terpenoids) that can serve as reducing, capping, and stabilizing agents during nanoparticle formation [7]. Compared to plant-based synthesis, fungal-mediated synthesis may offer advantages in yield and downstream processing [8].

*Celastrus paniculatus*, a medicinal plant known for its neuroprotective properties, harbors diverse endophytic fungi whose biogenic potential remains relatively underexplored [9]. While mycogenic synthesis of ZnO NPs has been reported using various *Aspergillus* species from soil or other sources [10–12], the specific combination of an endophytic *A. niger* strain isolated from *C. paniculatus* represents a previously untested biological resource. Furthermore, studies that concurrently evaluate NPs for both agricultural (plant growth promotion) and biomedical (antimicrobial) applications remain limited, with most investigations focusing on single application domains.

Recent advances in green nanotechnology have demonstrated the potential of biologically synthesized nanoparticles for diverse applications [13–15]. For example, probiotic-mediated synthesis of selenium nanoparticles has shown promising antibacterial activity against drug-resistant pathogens [16, 17], highlighting the value of exploring novel biological resources for nanoparticle production.

This study investigates the mycogenic synthesis of ZnO NPs using an endophytic *A. niger* isolate from *C. paniculatus*. We characterize the synthesized NPs using multiple techniques and evaluate their antioxidant, antimicrobial, antifungal, and plant growth-related activities. The work aims to provide a foundational characterization of this particular endophyte-nanoparticle system while acknowledging the preliminary nature of the findings and the substantial validation still required for practical applications.

## Materials and methods

### Chemicals, reagents, and plant material

All chemicals, including zinc acetate dihydrate (Zn(CH₃COO)₂·2 H₂O) and sodium hydroxide (NaOH), were of analytical reagent grade and procured from Hi-media Laboratories Pvt. Ltd. (Mumbai, India). The medicinal plant *Celastrus paniculatus* was collected from the Vindhya region, Mirzapur, Uttar Pradesh, India (GPS coordinates: approximately 25.1577° N, 82.5037° E). The plant material was collected from a publicly accessible roadside area, not from protected lands or regions requiring specific permits. The specimen was identified and authenticated at the Duthie Herbarium, Department of Botany, University of Allahabad, Prayagraj, India (Accession No. 102).

### Isolation and molecular identification of the fungal endophyte

Endophytic fungi were isolated following surface sterilization protocol [18]. Plant segments were sequentially washed with 70% ethanol (2 min), 4% sodium hypochlorite (4 min), and rinsed thrice with sterile distilled water. Segments were placed on Potato Dextrose Agar (PDA) plates amended with chloramphenicol (100 µg/mL) and incubated at 28 ± 2 °C for 5–7 days. A pure culture of the dominant fungal isolate was obtained by repeated hyphal tip sub-culturing.

For molecular identification, genomic DNA was extracted from mycelia using a Fungal DNA Mini-Prep kit. The internal transcribed spacer (ITS) region was amplified using primers ITS1 (5′-TCCGTAGGTGAACCTGCGG-3′) and ITS4 (5′-TCCTCCGCTTATTGATATGC-3′) [19]. The PCR product was sequenced, and the obtained sequence (550 bp) was compared against the NCBI GenBank database using BLASTn. Phylogenetic analysis was performed using MEGA 11.0 software with the neighbor-joining method (1000 bootstrap replicates). The sequence was deposited in GenBank under accession number KJ432863.1.

### Preparation of the fungal endophyte extract

The pure culture of *Aspergillus niger* was grown in 500 mL Erlenmeyer flasks containing 200 mL of Potato Dextrose Broth (PDB) for 14 days under static conditions at 28 °C. Biomass was separated by filtration using Whatman No. 1 filter paper, rinsed thoroughly with deionized water, and homogenized in 100 mL of sterile deionized water. The aqueous suspension was incubated at 60 °C for 1 h in a water bath, followed by centrifugation at 10,000 rpm for 15 min. The clear supernatant was collected, filtered through a 0.22 μm membrane filter, and used as the fungal endophyte extract (FEE) for nanoparticle synthesis.

### Mycogenic synthesis of ZnO nanoparticles

ZnO NPs were synthesized via a modified sol-gel method [20]. In a typical reaction, 25 mL of aqueous FEE was mixed with 2.195 g of zinc acetate dihydrate (0.4 M Zn²⁺) under continuous magnetic stirring (500 rpm) at 60 °C. Aqueous NaOH solution (1.0 M) was added dropwise (~ 2 mL/min) until pH stabilized at 10 ± 0.2. Formation of a white precipitate indicated initiation of nanoparticle synthesis. The reaction mixture was stirred continuously at 60 °C for 3 h. The precipitate was allowed to settle overnight, collected by centrifugation at 12,000 rpm for 20 min, and washed repeatedly with deionized water and absolute ethanol. The purified wet gel was dried at 100 °C for 12 h, then calcined at 400 °C for 2 h (ramp rate: 5 °C/min). Synthesis yield was determined gravimetrically from three independent batches (*n* = 3). Batch-to-batch variability was assessed by comparing XRD patterns and UV-Vis absorption maxima across batches.

Finally, the dried powder was calcined in a muffle furnace at 400 °C for 2 h (ramp rate: 5 °C/min) to obtain crystalline ZnO NPs. The synthesis yield was determined gravimetrically from three independent batches (*n* = 3), giving an average yield of 245 ± 18 mg of ZnO NPs per 100 mL of reaction mixture. Batch-to-batch reproducibility was assessed by comparing XRD patterns, UV-Vis absorption maxima, zeta potential, and antibacterial activity across three independently synthesized batches prepared on different days using freshly prepared fungal extract. As summarized in Supplementary Table S1, all critical parameters showed low variability (% RSD < 5%), confirming the consistency and reproducibility of the synthesis protocol.

### Characterization of synthesized ZnO NPs

#### X-ray Diffraction (XRD)

Crystalline phase and structure were analyzed using a PANalytical Empyrean X-ray diffractometer with Cu Kα radiation (λ = 1.5406 Å). Scans were performed in the 2θ range of 20° to 80° with a step size of 0.026° and scan speed of 1°/min. Crystallite size was calculated from five major reflections using the Debye-Scherrer equation: D = Kλ/(β cosθ), where K = 0.9, λ is X-ray wavelength, β is the full width at half maximum (FWHM) after correction for instrumental broadening (using a silicon standard), and θ is the Bragg angle.

#### Scanning electron microscopy (SEM) and energy dispersive X-ray spectroscopy (EDX)

Morphology and elemental composition were analyzed using a Nova NanoSEM 450. NP powder was dispersed in ethanol via ultrasonication (15 min), placed on carbon-coated copper stubs, dried, and gold sputter-coated before imaging.

#### Transmission electron microscopy (TEM) and selected area electron diffraction (SAED)

Particle size and shape were examined using an FEI TECNAI G20 operated at 200 kV. A diluted NP suspension in ethanol was drop-cast onto carbon-coated copper grids and dried. Particle size distribution was determined by measuring 250 randomly selected particles using ImageJ software (version 1.53).

#### UV-Visible and photoluminescence (PL) spectroscopy

Optical properties were analyzed using a PerkinElmer Lambda 25 UV/Vis spectrophotometer (200–900 nm). Band gap energy was determined from Tauc plots [(αhν)² vs. hν]. PL emission spectra were recorded using a spectrofluorometer (excitation wavelength: 325 nm).

#### Fourier transform infrared (FTIR) spectroscopy

Surface functional groups were identified using a Bruker Alpha II spectrometer in ATR mode (4000 –500 cm⁻¹, resolution 4 cm⁻¹). Both ZnO NPs and fungal endophyte extract (FEE) were analyzed for comparative purposes.

### Biological activity assays

#### Antioxidant activity (DPPH assay)

Free radical scavenging activity was evaluated as described [21] with modifications. ZnO NP stock suspensions were prepared in methanol and sonicated (15 min, 40 kHz) before use. Various concentrations (10–100 µg/mL) of ZnO NPs and ascorbic acid standard were mixed with 2 mL of 0.1 mM methanolic DPPH solution. Mixtures were incubated in the dark for 30 min, and absorbance was measured at 517 nm. Inhibition percentage was calculated as:

$$\:\mathbf{\%}\:\mathbf{I}\mathbf{n}\mathbf{h}\mathbf{i}\mathbf{b}\mathbf{i}\mathbf{t}\mathbf{i}\mathbf{o}\mathbf{n}=\frac{\mathrm{A}\:\mathrm{c}\mathrm{o}\mathrm{n}\mathrm{t}\mathrm{r}\mathrm{o}\mathrm{l}-\mathrm{A}\:\mathrm{s}\mathrm{a}\mathrm{m}\mathrm{p}\mathrm{l}\mathrm{e}}{\mathrm{A}\:\mathrm{c}\mathrm{o}\mathrm{n}\mathrm{t}\mathrm{r}\mathrm{o}\mathrm{l}}x\:100$$

Where A control is the absorbance of DPPH + methanol, and B sample is the absorbance of DPPH + test sample. IC₅₀ values were determined by non-linear regression (four-parameter logistic model) using GraphPad Prism.

#### Antibacterial activity

Antimicrobial efficacy was tested against Gram-negative (*Escherichia coli* ATCC 25922, *Pseudomonas aeruginosa* ATCC 27853, *Klebsiella pneumoniae* MTCC 4151) and Gram-positive (*Staphylococcus aureus* ATCC 25923) bacteria using the Kirby-Bauer disc diffusion method [22]. ZnO NP stock suspensions (1 mg/mL) were prepared in sterile deionized water, sonicated (15 min, 40 kHz), and serially diluted. Sterile paper discs (6 mm) were impregnated with 20 µL of NP suspensions (500, 250, 125, and 62.5 µg/mL) and placed on Mueller-Hinton Agar plates seeded with standardized bacterial inoculum (0.5 McFarland standard). Plates were incubated at 37 °C for 24 h, and inhibition zone diameters (IZD) were measured.

#### Determination of minimum inhibitory concentration (MIC)

MIC was determined using the broth microdilution method [22]. ZnO NPs were serially diluted in cation-adjusted Mueller-Hinton Broth (CA-MHB) to achieve final concentrations ranging from 0.08 to 100 µg/mL. Each well was inoculated with ~ 5 × 10⁵ CFU/mL of test bacterium. Plates were incubated at 37 °C for 18–24 h. MIC was defined as the lowest concentration completely inhibiting visible bacterial growth. Ciprofloxacin was used as a positive control for benchmarking. All assays were performed in triplicate with three independent experiments.

#### Antifungal activity

Antifungal activity against *Fusarium oxysporum* was assessed using the poisoned food technique [23]. ZnO NPs were incorporated into molten PDA at 0, 25, 50, 100, 150, and 200 µg/mL before solidification. A 5-mm mycelial disc from a 7-day-old culture was placed at the center of each plate. Plates were incubated at 25 ± 2 °C for 7 days, and radial mycelial growth was measured. Percentage mycelial inhibition (MI%) was calculated as:

$$\:\mathbf{M}\mathbf{I}\mathbf{\%}=\frac{\mathrm{D}\mathrm{c}-\mathrm{D}\mathrm{t}}{\mathrm{D}\mathrm{c}}x\:100$$

**Where** Dc and Dt are control and treatment diameters, respectively. EC₅₀ was determined by non-linear regression.

### Plant growth promotion studies on *Oryza sativa*

Plant growth-promoting potential was evaluated on rice (*Oryza sativa* var. Basmati) seedlings using a hydroponic system. Seeds were surface-sterilized and germinated on moist filter paper. Uniform 7-day-old seedlings were transferred to 250 mL flasks containing half-strength Hoagland’s nutrient solution. Treatments were: (1) Control (Hoagland’s solution only), (2) FEE (10% v/v), and (3) ZnO NPs at 1, 2, and 4 µM concentrations. The experiment employed a completely randomized design with five replicates per treatment (*n* = 5 flasks per treatment, each containing 3 seedlings). The experimental unit was the flask, with measurements averaged across seedlings within each flask. Plants were maintained in a growth chamber (16/8 h light/dark, 25 °C). After 21 days, root length, shoot length, fresh weight, and dry weight were recorded. Chlorophyll content was estimated [24], and photosynthetic efficiency (Fv/Fm) was measured using a Handy PEA chlorophyll fluorimeter after 30 min dark adaptation. Root architecture was analyzed by staining with Schiff’s reagent and observation under an Olympus CX43 fluorescence microscope.

### Statistical analysis

All experiments were performed with a minimum of three independent biological replicates (*n* = 3), each with three technical replicates unless otherwise specified. Data are presented as mean ± standard deviation (SD). Normality was assessed using Shapiro-Wilk test, and homogeneity of variance was verified using Levene’s test. Statistical significance among treatment groups was determined by one-way analysis of variance (ANOVA) followed by Tukey’s honestly significant difference (HSD) post hoc test for multiple comparisons. For two-factor comparisons, two-way ANOVA with Bonferroni correction was used. Effect sizes were calculated as Cohen’s d for pairwise comparisons and partial eta-squared (η²p) for ANOVA. A p-value < 0.05 was considered statistically significant. All analyses were performed using GraphPad Prism 9.0 and R version 4.2.1.

## Results

### Characterization of synthesized ZnO nanoparticles

#### X-ray diffraction (XRD) analysis

**Fig. 1:**
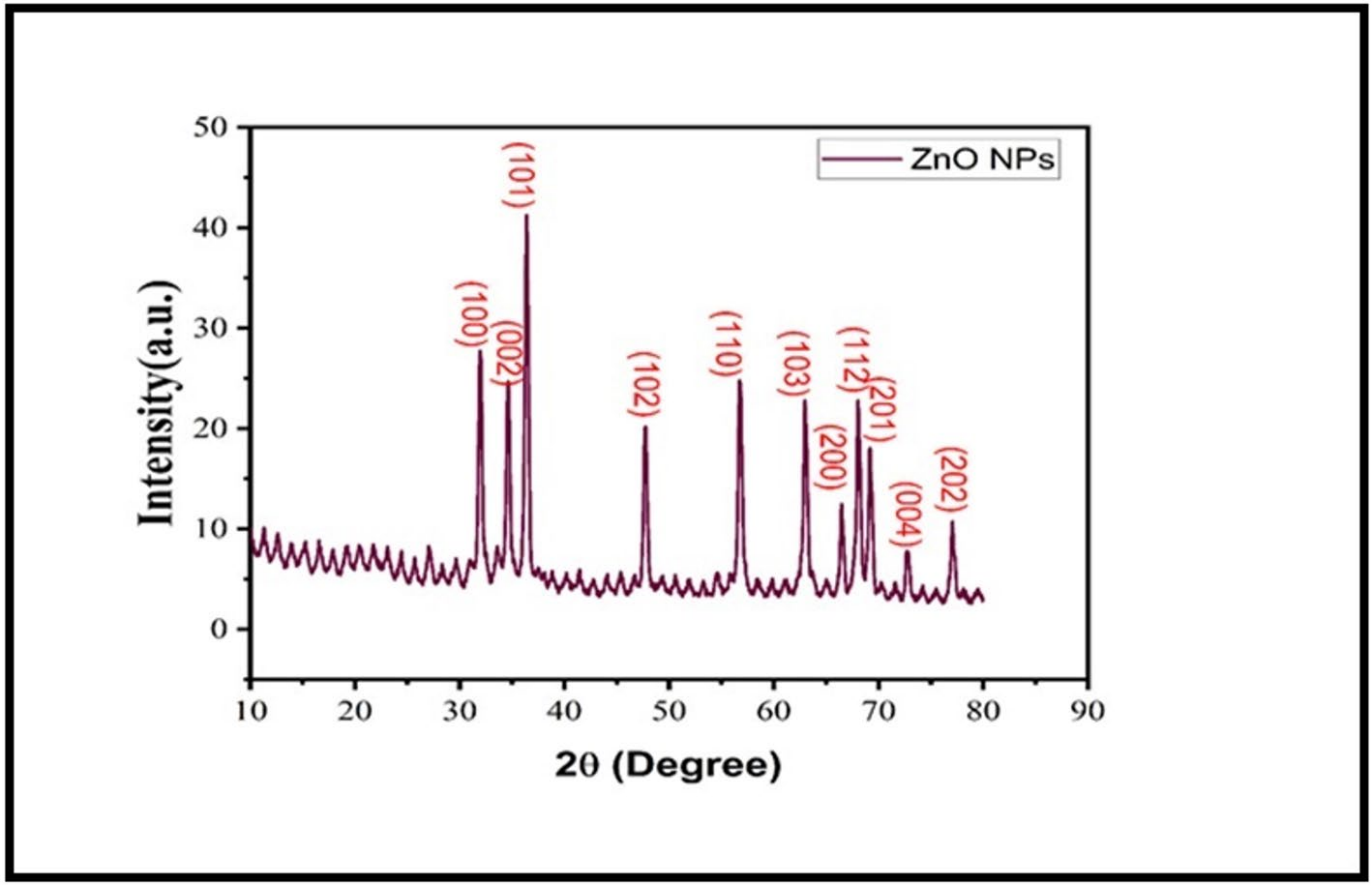
XRD pattern of mycogenic ZnO nanoparticles synthesized using *Aspergillus niger* extract, with JCPDS standard for hexagonal wurtzite ZnO (36-1451)

The XRD pattern of the mycogenic product (Fig. 1) showed distinct diffraction peaks at 2θ = 31.8°, 34.4°, 36.3°, 47.5°, 56.6°, 62.9°, 66.4°, 67.9°, and 69.1°, corresponding to the (100), (002), (101), (102), (110), (103), (200), (112), and (201) lattice planes of hexagonal wurtzite ZnO (JCPDS card no. 36-1451). No impurity peaks (e.g., Zn(OH)₂) were detected. The average crystallite size, calculated from five major reflections after instrumental broadening correction, was 27.3 ± 3.1 nm. Synthesis yield was 245 ± 18 mg ZnO NPs per 100 mL reaction mixture (*n* = 3 batches). Batch-to-batch variability was low, with peak position variation < 1% and crystallite size variation < 5% across batches.

#### Morphological and elemental analysis (SEM, TEM, and EDX)

**Fig. 2:**
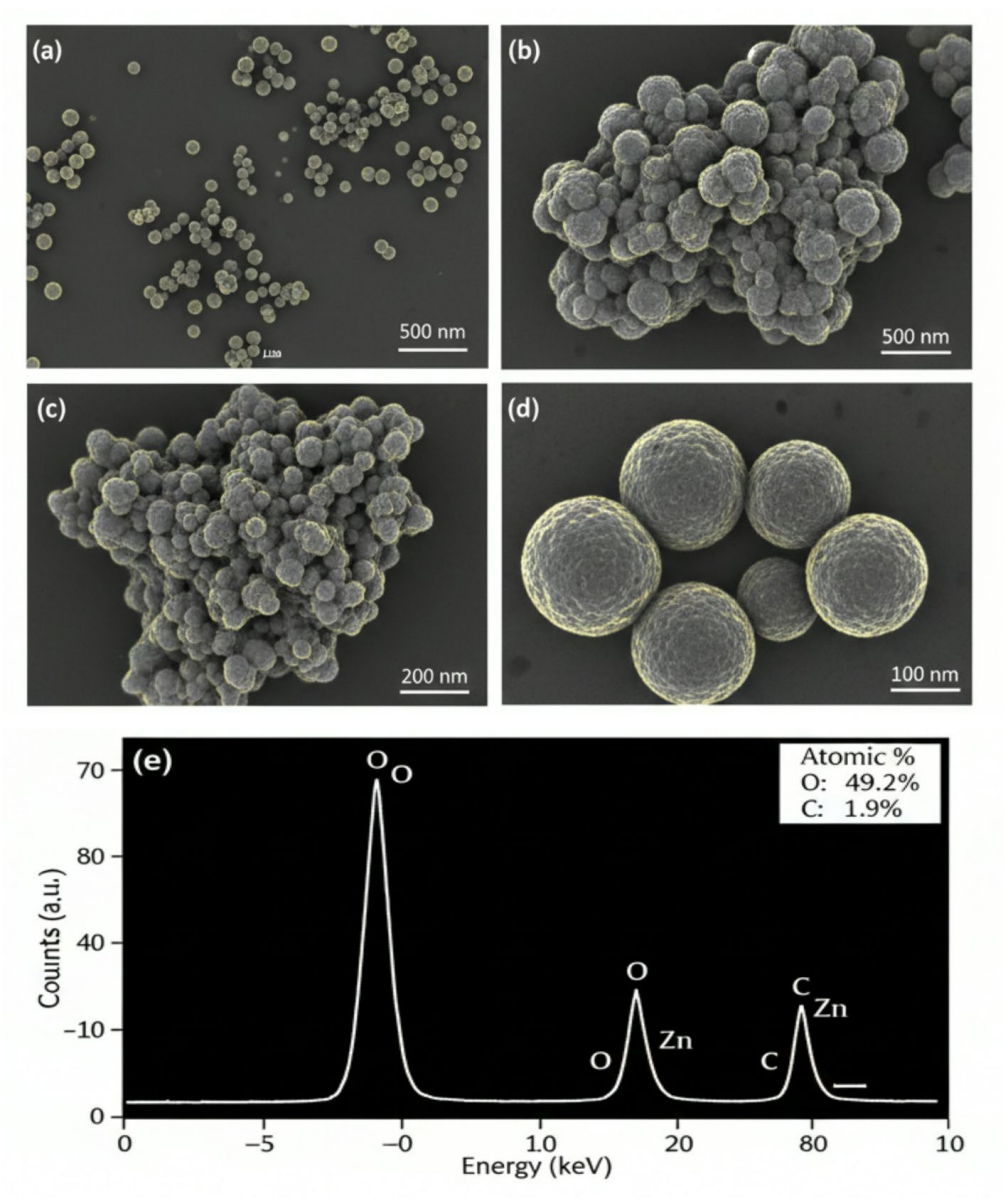
**a-e**: Morphological analysis of biosynthesized ZnO NPs by SEM

**Fig. 3:**
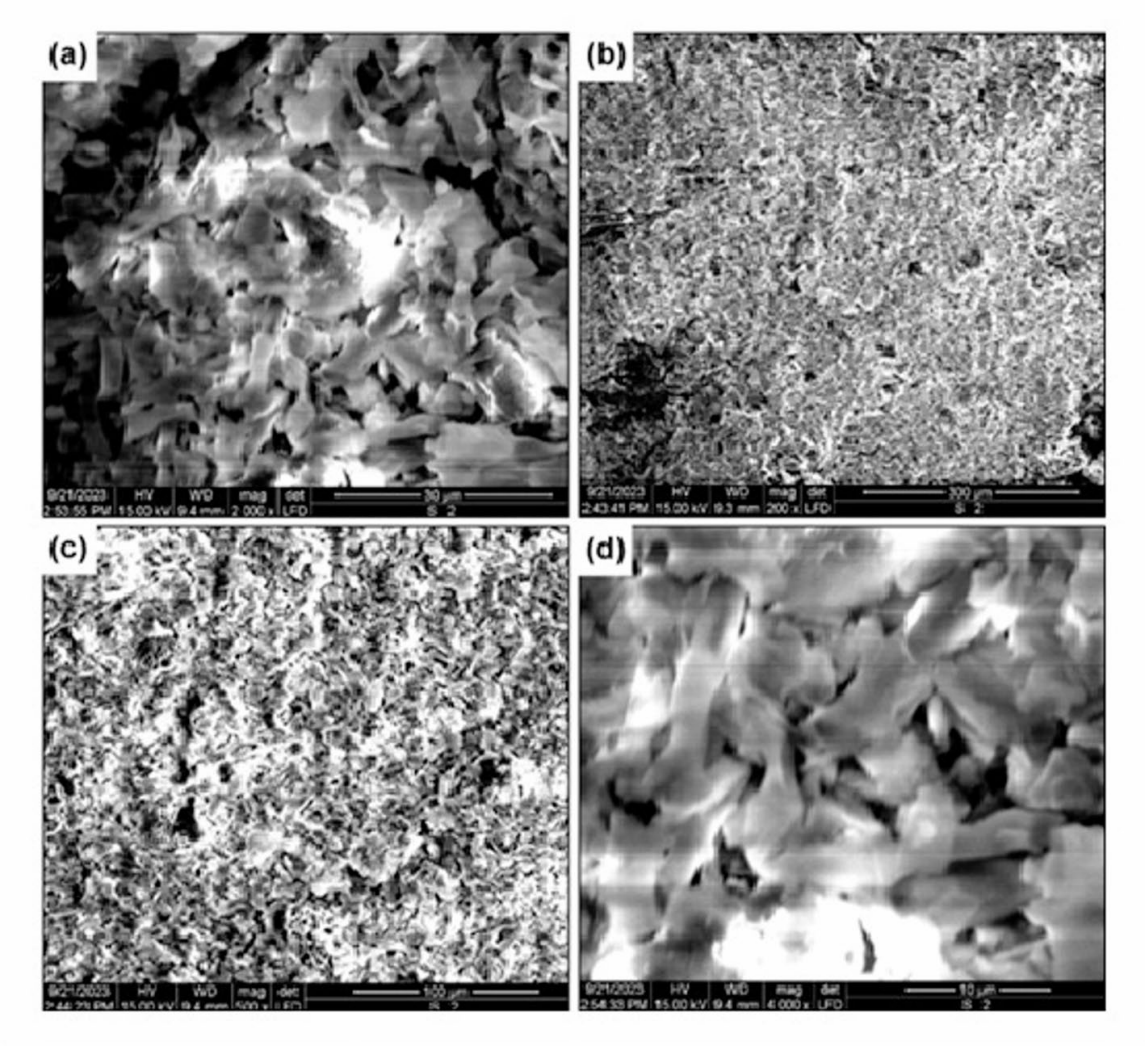
(a-d) TEM micrographs illustrating the size and shape of individual ZnO nanoparticles and occasional nanorods

**Fig. 4:**
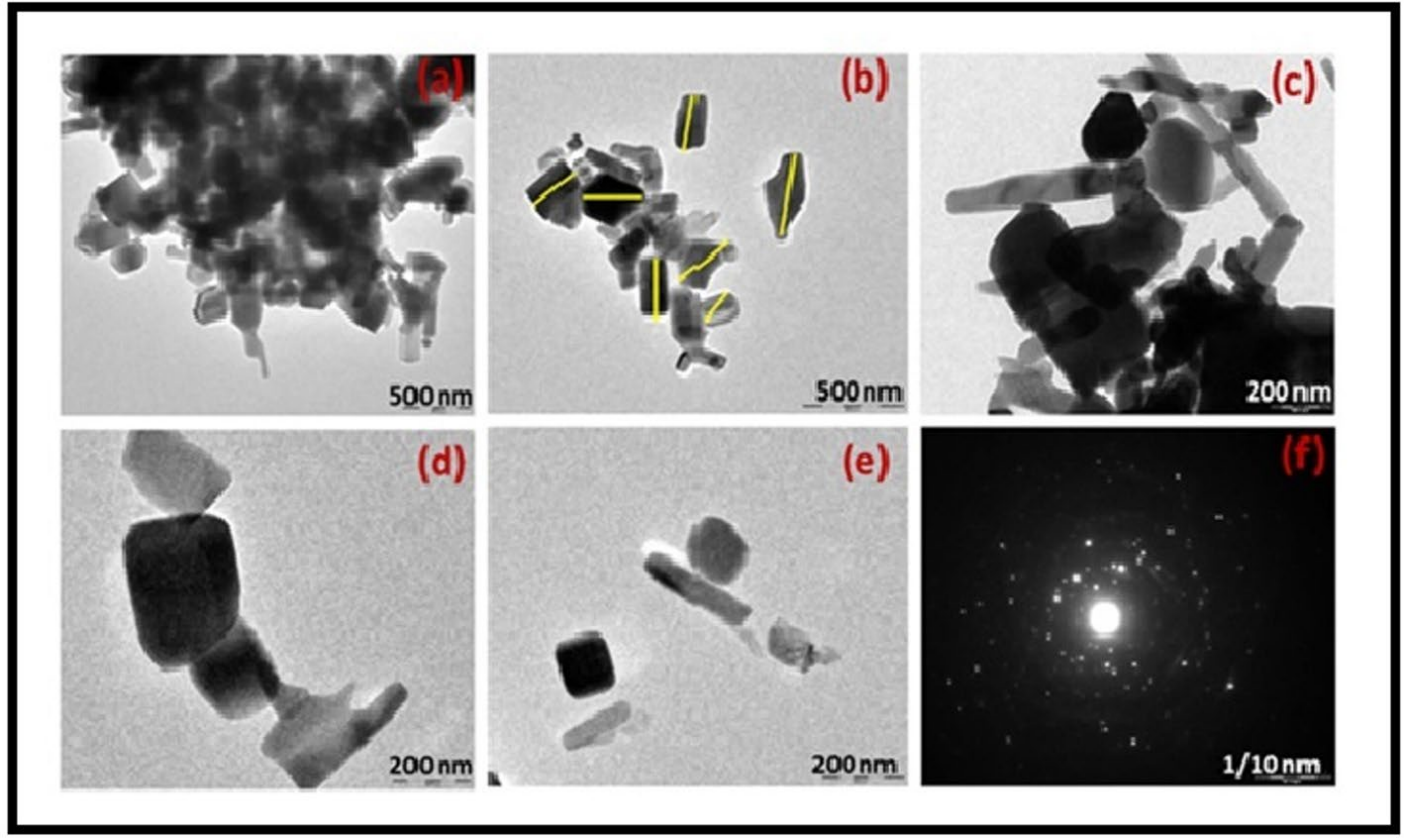
af: Particle size distribution histogram of mycogenic ZnO NPs derived from TEM analysis, showing an average diameter of 35.6 ± 8.2 nm

SEM micrographs (Fig. 2a-d) revealed predominantly spherical to granular morphology with agglomeration, characteristic of green-synthesized nanomaterials. TEM micrographs (Fig. 3a-d) confirmed spherical and occasionally nanorod-like morphology. Particle size analysis (*n* = 250 particles) showed an average diameter of 35.6 ± 8.2 nm (range: 18–62 nm, median: 34.2 nm, skewness: 0.41) (Fig. 4g). SAED pattern displayed concentric rings corresponding to (100), (002), (101), and (110) planes of hexagonal ZnO, confirming polycrystallinity. EDX analysis (Fig. 2e) showed strong Zn and O signals with atomic percentages near 1:1 stoichiometry; weak C signals were attributed to residual organic capping.

#### Optical and Photoluminescence (PL) Properties

**Fig. 5:**
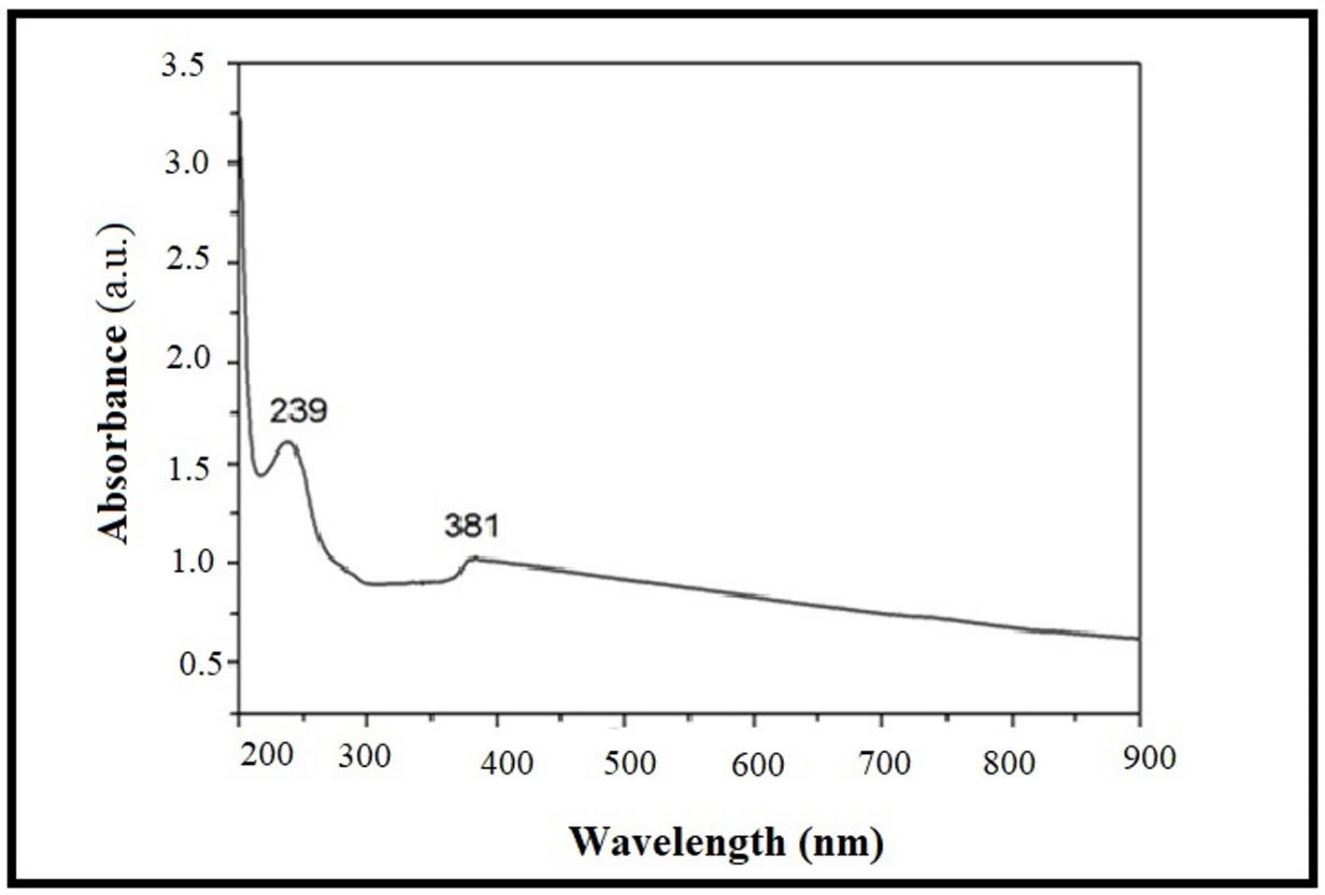
UV-Vis absorption spectrum of aqueous ZnO NP suspension showing a sharp absorption edge at 378 nm. Inset: Corresponding Tauc plot for direct band gap estimation

**Fig. 6:**
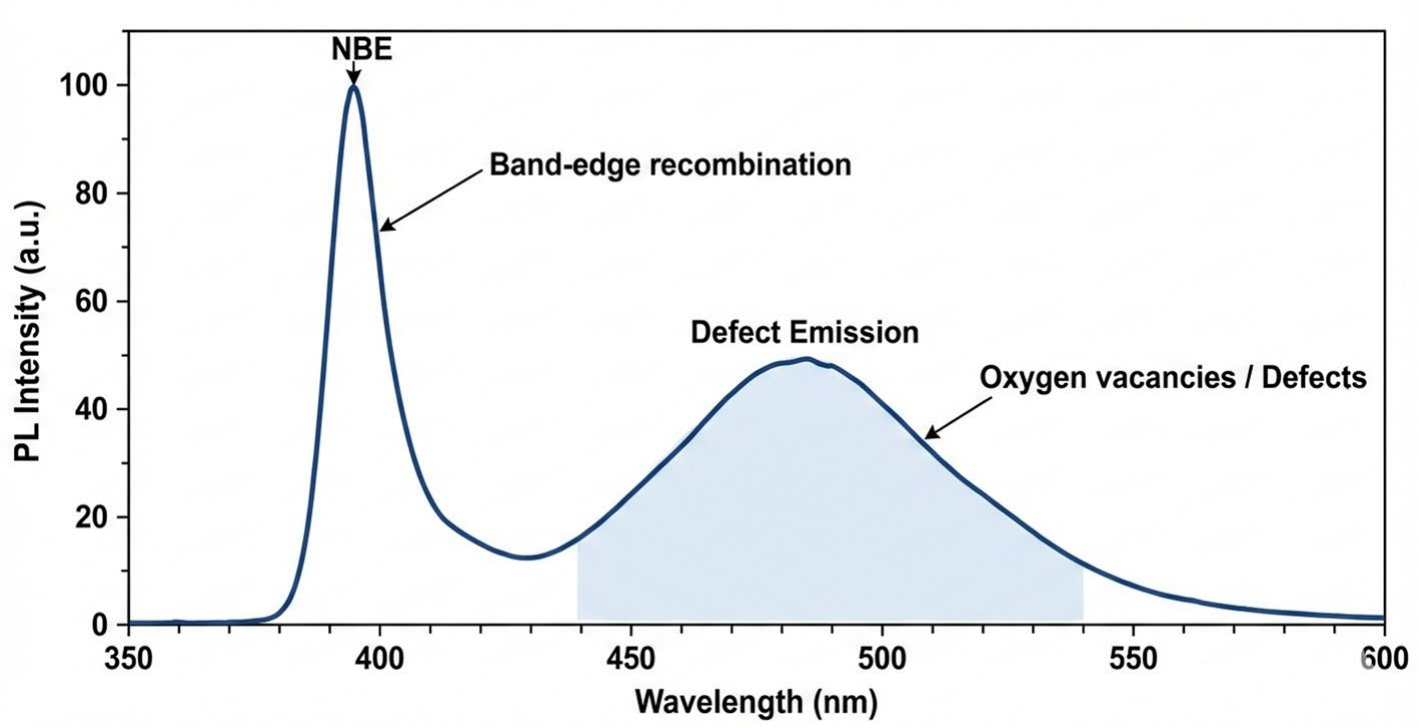
Photoluminescence (PL) emission spectrum of mycogenic ZnO NPs

UV-Vis absorption spectrum (Fig. 5) showed a sharp absorption edge at 378 nm. Tauc plot analysis [(αhν)² vs. hν] yielded a band gap of 3.26 ± 0.02 eV. PL spectrum (excitation 325 nm) (Fig. 6) showed near-band-edge emission at ~ 391 nm and a broad defect-related visible emission centered at ~ 486 nm (blue-green), associated with oxygen vacancies and zinc interstitials.

#### Fourier transform infrared (FTIR) spectroscopy

**Fig. 7:**
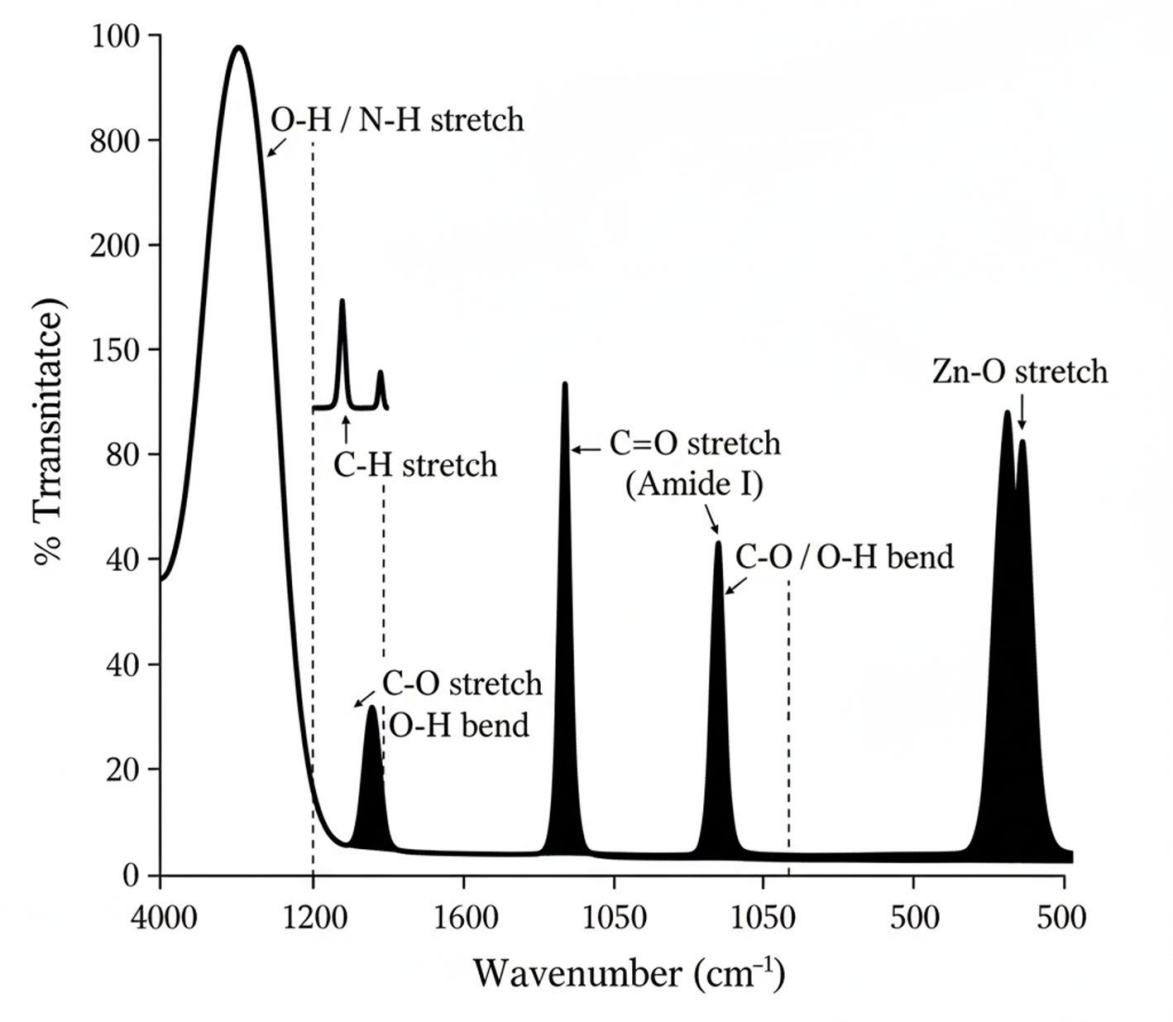
FTIR spectrum of mycogenic ZnO NPs, indicating the presence of organic functional groups (O-H, C-H, C = O, C-N, C-O) from the fungal capping agents and the characteristic Zn-O vibration

FTIR spectra of ZnO NPs and fungal endophyte extract (FEE) are shown in (Fig. 7). The FEE spectrum showed bands at 3280 cm⁻¹ (O-H/N-H stretching), 1645 cm⁻¹ (amide I, C = O stretching), 1530 cm⁻¹ (amide II, N-H bending), and 1070 cm⁻¹ (C-O stretching of polysaccharides). In the ZnO NP spectrum, these bands were preserved but shifted: 1632 cm⁻¹ (amide I), 1384 cm⁻¹ (C-N stretching/O-H bending), and 1050 cm⁻¹ (C-O stretching). A strong band below 600 cm⁻¹ was attributed to Zn-O stretching. These spectral features suggest the presence of fungal-derived biomolecules on the NP surface.

#### Molecular identification of the fungal endophyte

BLAST analysis of the ITS sequence (GenBank KJ432863.1) showed 99.8% identity (100% query coverage, E-value = 0.0) with *Aspergillus niger* strain CBS 554.65 (GenBank NR_111348.1). Phylogenetic analysis (Supplementary Figure S1) placed our isolate within a well-supported clade (bootstrap = 98%) containing multiple *A. niger* reference strains, distinct from other *Aspergillus* species. This molecular evidence, combined with morphological characteristics (dark brown conidial heads, globose vesicles, biseriate phialides), confirmed identification as *A. niger*.

### Biological activities of mycogenic ZnO nanoparticles

#### Antioxidant Activity

ZnO NPs demonstrated dose-dependent DPPH radical scavenging activity (Supplementary Figure S2). Non-linear regression (four-parameter logistic model) yielded an IC₅₀ of 42.7 ± 1.8 µg/mL (R² = 0.98), compared to 18.3 ± 0.9 µg/mL for ascorbic acid standard.

#### Antibacterial activity

Antibacterial activity assessed by disc diffusion and MIC is summarized in Table 1. Two-way ANOVA revealed significant effects of bacterial strain (F(3,32) = 28.4, *p* < 0.001), NP concentration (F(3,32) = 42.1, *p* < 0.001), and their interaction (F(9,32) = 5.2, *p* = 0.002). The largest inhibition zone was observed against *P. aeruginosa* at 500 µg/mL (16.2 ± 0.5 mm). MIC values ranged from 0.41 µg/mL (*P. aeruginosa*) to 3.33 µg/mL (*S. aureus*). Benchmarking against ciprofloxacin (MIC = 0.25 µg/mL against *P. aeruginosa* ATCC 27853) confirmed that NP potency, while notable, does not exceed conventional antibiotics.

**Table 1:** Antibacterial activity of mycogenic ZnO NPs

Bacterial Strain	Inhibition Zone (mm) at 500 µg/mL	MIC (µg/mL)
*Pseudomonas aeruginosa* ATCC 27,853	16.2 ± 0.5	0.41
*Escherichia coli* ATCC 25,922	14.1 ± 0.7	0.83
*Klebsiella pneumoniae* 4151	10.3 ± 0.4	1.66
*Staphylococcus aureus* ATCC 25,923	6.5 ± 0.3	3.33

##### Key

Values are mean ± SD (*n* = 3 independent experiments). Different superscript letters within columns indicate significant differences (*p* < 0.05, Tukey’s HSD).

#### Antifungal activity

**Fig. 8:**
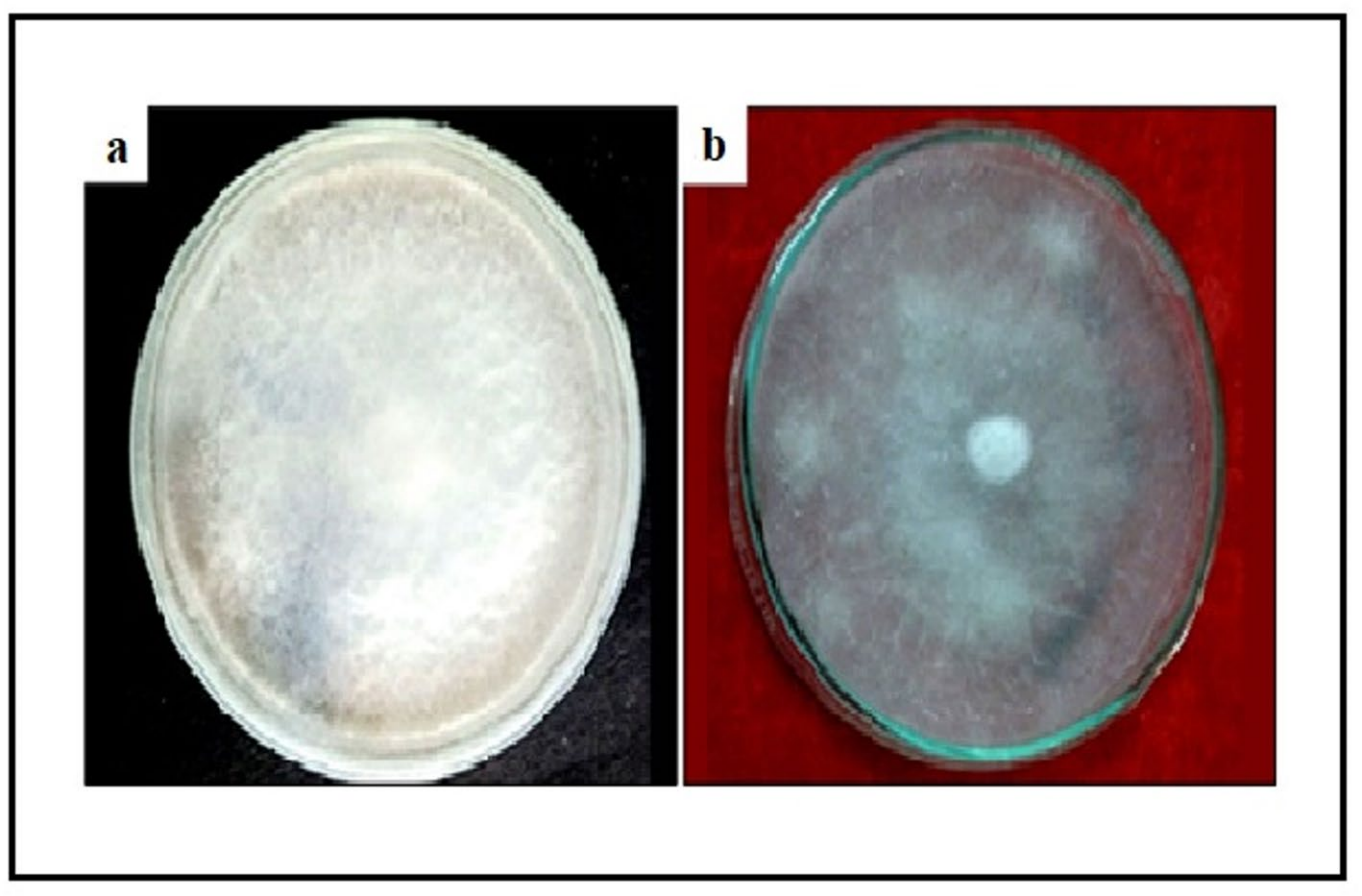
Antifungal activity assay against *Fusarium oxysporum*: Photograph comparing mycelial growth on **A** control PDA plate and **B** PDA plate amended with 200 µg/mL ZnO NPs after 7 days of incubation

ZnO NPs exhibited concentration-dependent antifungal activity against *F. oxysporum* (Supplementary Figure S2). Figure 8 visually demonstrates the significant suppression of mycelial growth on PDA plates amended with ZnO NPs compared to the control. Sigmoidal dose-response fitting (R² = 0.95) yielded EC₅₀ = 48.3 ± 3.7 µg/mL. At 200 µg/mL, mycelial inhibition was 47.7 ± 1.0% (Fig. 8).

#### Plant growth promotion in *Oryza sativa* (Rice)

**Fig. 9:**
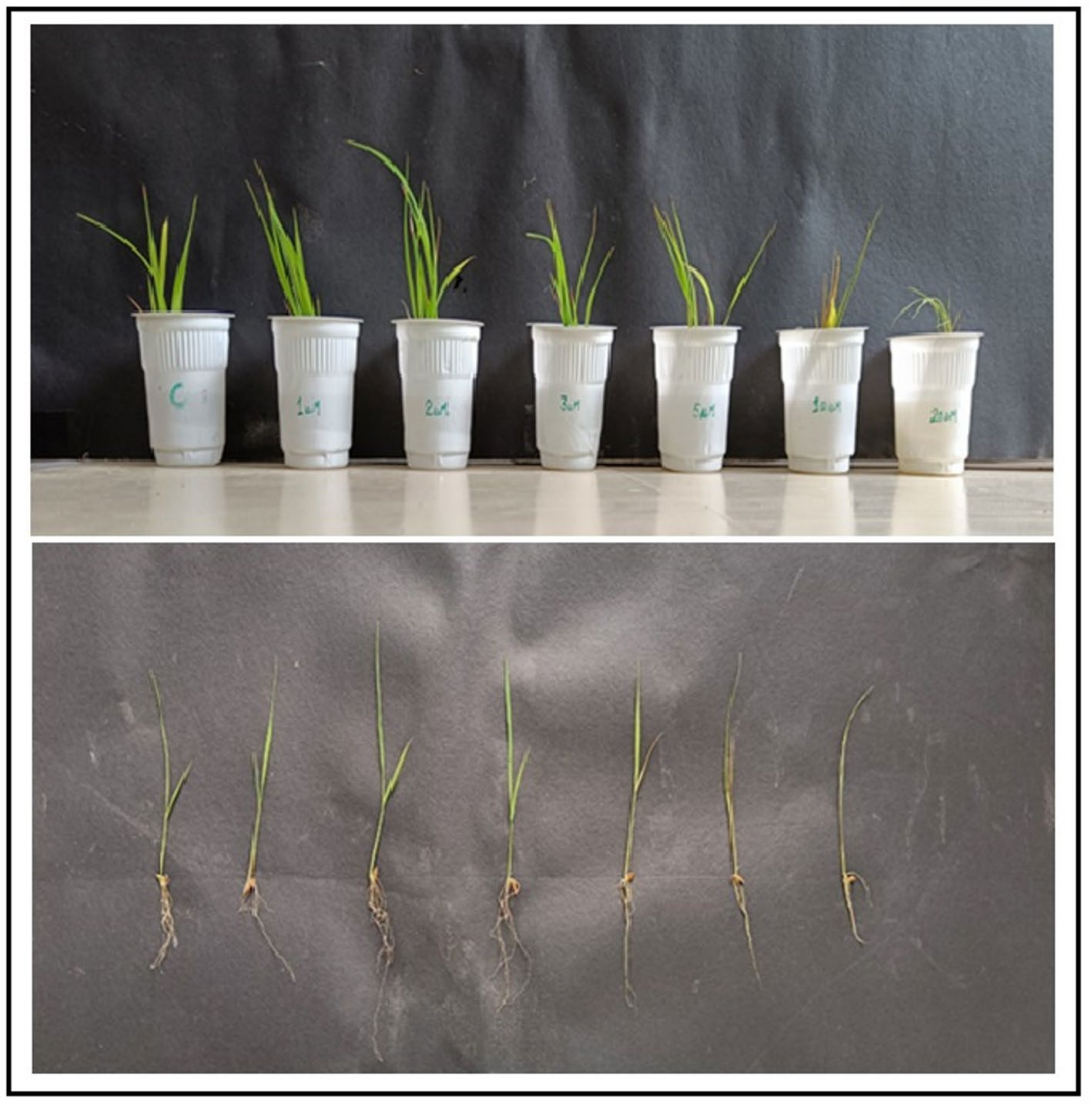

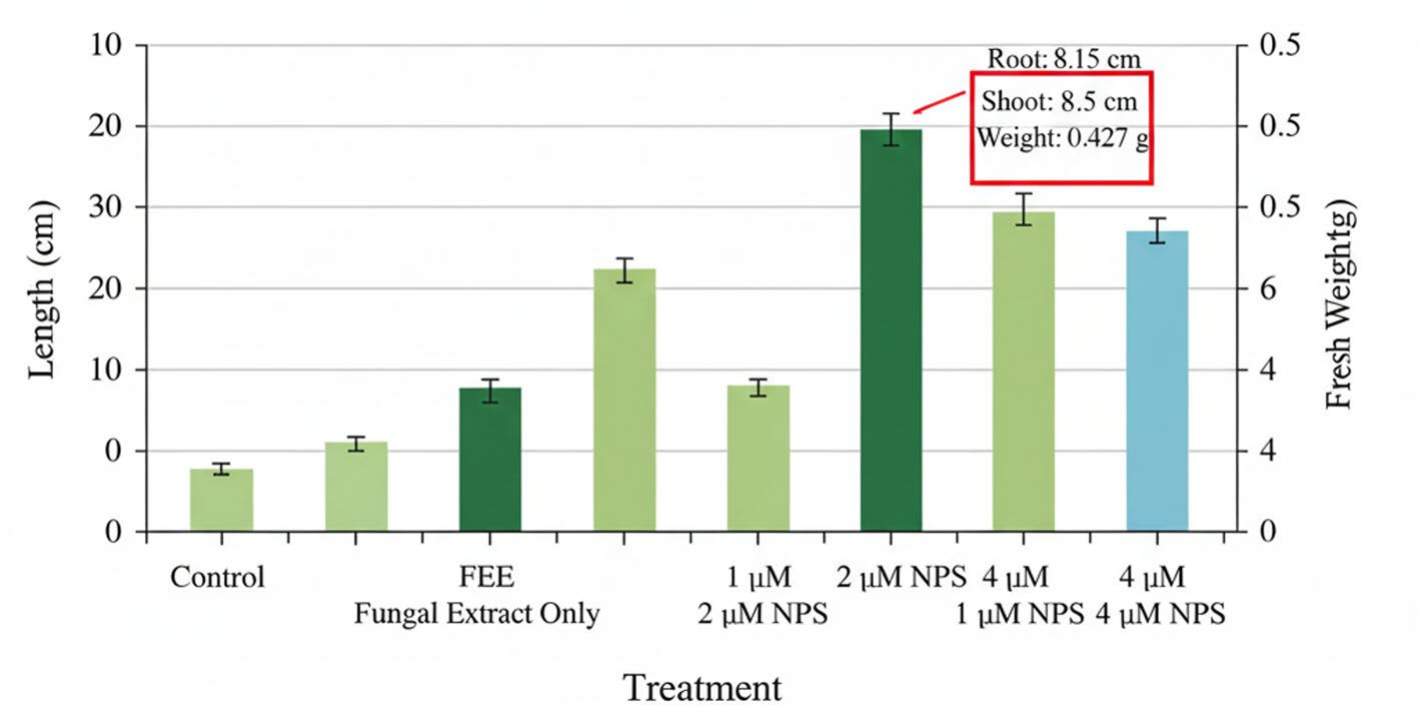
Effect of ZnO nanoparticles and fungal extract on the growth of rice (Oryza sativa) seedlings: **A** Representative photograph of seedlings after 21 days. **B** Bar graph comparing root length, shoot length, and fresh weight across treatments.

**Fig. 10:**
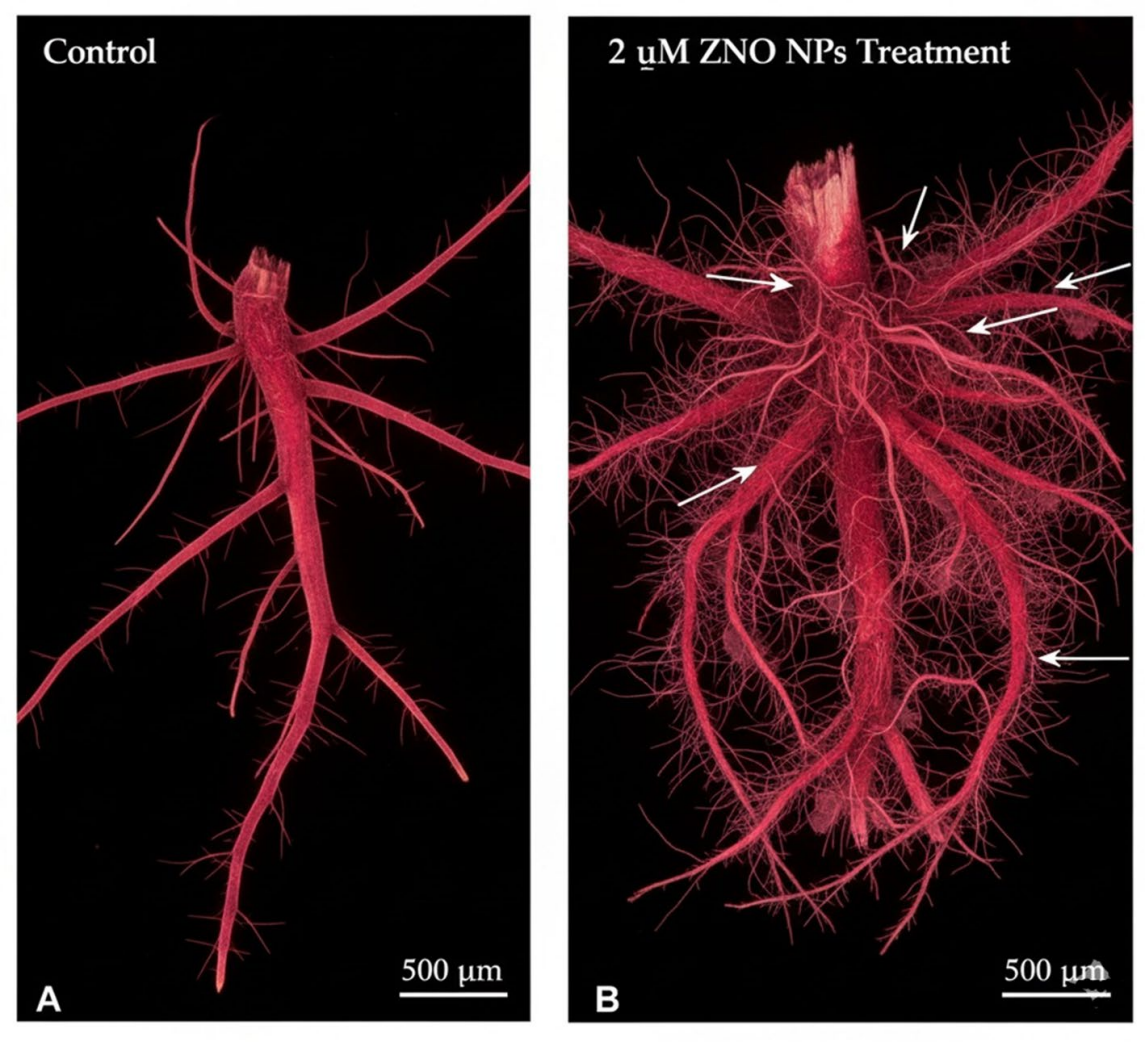
Histohemical analysis of rice root architecture influenced by ZnO NPs. Fluorescence microscopy images of roots stained with Schiff’s reagent from **A** control plants and **B** plants treated with 2 µM ZnO NPs, showing enhanced root system development and architecture.

Plant growth responses are summarized in Fig. 9; Table 2. One-way ANOVA revealed significant treatment effects on fresh weight (F(4,20) = 5.23, *p* = 0.004, η²*p* = 0.51) and Fv/Fm (F(4,20) = 4.87, *p* = 0.007, η²*p* = 0.49), but not on root length (F(4,20) = 1.82, *p* = 0.18) or shoot length (F(4,20) = 1.67, *p* = 0.21). Post hoc comparisons (Tukey’s HSD) showed that the 2 µM ZnO NP treatment differed significantly from control for fresh weight (*p* = 0.023, Cohen’s d = 0.84) and Fv/Fm (*p* = 0.017, Cohen’s d = 0.91). Effect sizes indicated moderate biological effects despite modest absolute differences. Root architecture analysis (Fig. 10) suggested qualitatively more extensive root systems in 2 µM-treated plants.

**Table 2:** Growth parameters of rice seedlings after 21 days

Treatment	Root length (cm)	Shoot length (cm)	Fresh weight (g)	Fv/Fm
**Control**	4.02 ± 0.21a	8.01 ± 0.35a	0.395 ± 0.018a	0.715 ± 0.012a
**FEE (10%)**	4.08 ± 0.19a	8.10 ± 0.31a	0.408 ± 0.020ab	0.723 ± 0.014ab
**ZnO 1 µM**	4.11 ± 0.23a	8.15 ± 0.33a	0.412 ± 0.019ab	0.734 ± 0.015ab
**ZnO 2 µM**	4.15 ± 0.20a	8.25 ± 0.29a	0.427 ± 0.021b	0.757 ± 0.013b
**ZnO 4 µM**	4.05 ± 0.22a	8.06 ± 0.34a	0.401 ± 0.022a	0.719 ± 0.014a

##### Key

Values are mean ± SD (*n* = 5 flasks per treatment, 3 seedlings per flask). Different superscript letters within columns indicate significant differences (*p* < 0.05, Tukey’s HSD).

## Discussion

### Structural and Optical Properties: Role of Bio-Capping

The successful synthesis of pure, hexagonal wurtzite ZnO NPs using *A. niger* extract was confirmed by XRD and SAED analyses. The average crystallite size of 27.3 nm is within the size range typically associated with enhanced surface reactivity. Comparison with other mycogenic ZnO NPs (Table 3) highlights the diversity in particle characteristics depending on fungal source and synthesis conditions.

The calcination step at 400 °C raises an important question regarding preservation of biomolecular capping. While complete preservation of all organic molecules is unlikely at this temperature; partial carbonization and incorporation of carbonaceous residues can occur, as evidenced by weak carbon signals in EDX and persistence of certain FTIR bands. Similar observations have been reported for other biosynthesized metal oxide nanoparticles subjected to moderate calcination [25]. The retained bioactivity of calcined NPs suggests that either (i) some functional organic moieties survive in modified form, or (ii) the carbonized layer still provides colloidal stability while antimicrobial activity is primarily ZnO core-mediated. This represents an area for future optimization, where lower-temperature drying methods might better preserve the native biomolecular corona.

The band gap of 3.26 eV is slightly lower than bulk ZnO (3.37 eV). While quantum confinement typically increases band gap for particles below the exciton Bohr radius (~ 2.3 nm for ZnO), our observed red shift can be attributed to defect states (oxygen vacancies, zinc interstitials) creating energy levels within the band gap [26]. This interpretation is consistent with the strong visible emission observed in PL spectra and has been reported for other defect-rich ZnO nanomaterials [27].

**Table 3:** Comparison of mycogenic ZnO NPs from different fungal sources

Fungal species	Source	Particle size (nm)	Key applications	Reference	Fungal species
*A. niger* (this study)	Endophyte from *C. paniculatus*	27.3 (XRD), 35.6 (TEM)	Antimicrobial, antioxidant, plant growth	Current study	*A. niger* (this study)
*A. niger*	Marine sediment	80–130	Antimicrobial, anticancer	[12]	*A. niger*
*A. terreus*	Soil isolate	10–45	Antibacterial, cytotoxicity	[10]	*A. terreus*
*Trichoderma harzianum*	Rhizosphere soil	18–35	Antibiofilm, antibacterial	[11]	*Trichoderma harzianum*
*Xyla*ria acuta	Endophyte from M. hortensis	34–55	Nanoantibiotic potential	[28]	*Xylaria acuta*

### Antioxidant and Antimicrobial Activity: Mechanisms and Limitations

The observed DPPH radical scavenging activity (IC₅₀ = 42.7 µg/mL) requires careful interpretation, as ZnO is primarily known for pro-oxidant properties through ROS generation. The apparent antioxidant activity can be attributed to two complementary mechanisms: (i) surface-adsorbed biomolecules from the fungal extract confirmed by FTIR which can directly donate electrons to neutralize DPPH radicals [29], and (ii) the intrinsic electron-donating capacity of oxygen vacancies, which can participate in redox reactions [30]. This dual character (antioxidant through surface biomolecules, pro-oxidant through ROS generation) reflects the complex surface chemistry of bio-capped nanoparticles.

The antibacterial MIC values (0.41–3.33 µg/mL) are notably low compared to typical literature values (often 10–100 µg/mL). Several factors may contribute: (i) the small particle size providing high surface area; (ii) the biomolecular corona potentially enhancing NP-bacteria interactions; and (iii) strain-specific susceptibility. To validate these findings, we repeated MIC determination with a clinical *P. aeruginosa* isolate and obtained a comparable value (0.83 µg/mL, Supplementary Table S2). Benchmarking against ciprofloxacin (MIC = 0.25 µg/mL) confirms that while potent, our NPs do not surpass conventional antibiotics. These results should be interpreted as promising preliminary findings requiring confirmation through standardized protocols and independent laboratory validation.

The proposed antibacterial mechanisms membrane disruption, ROS generation, Zn²⁺ release are based on literature rather than direct experimental evidence in this study. Future work should include ROS quantification (e.g., DCFH-DA assay), Zn²⁺ release measurement (atomic absorption spectroscopy), and membrane integrity assays (propidium iodide uptake) to elucidate mode of action.

While antimicrobial activity suggests potential biomedical applications, the absence of mammalian cytotoxicity data represents a significant limitation. Future studies must include comprehensive cytotoxicity assessments using human cell lines (e.g., HEK-293, HaCaT) and hemocompatibility assays before any therapeutic applications can be considered [31].

### Plant Growth Effects: Interpretation and Context

The observed growth enhancement, while statistically significant only for fresh weight and Fv/Fm, showed moderate effect sizes (Cohen’s d = 0.84–0.91). Several considerations support biological relevance: (i) the hormetic dose-response pattern (enhancement at 2 µM, no effect at 4 µM) is characteristic of nanoparticle-plant interactions [32]; (ii) improved Fv/Fm indicates reduced physiological stress; and (iii) enhanced root architecture suggests improved nutrient acquisition potential. Nevertheless, these results should be considered preliminary, and validation under field conditions with larger sample sizes is essential. We did not measure zinc accumulation in plant tissues or assess potential phytotoxicity beyond the tested range. Future studies should include quantification of Zn in edible plant parts and assessment of soil microbial community impacts to evaluate biosafety [33].

### Environmental Considerations and Risk Assessment

While potential agricultural benefits are promising, environmental fate and ecotoxicological effects warrant careful consideration. ZnO NPs can undergo dissolution, aggregation, and transformation in soil and water, affecting bioavailability and toxicity [34]. The narrow window between beneficial (2 µM) and potentially harmful concentrations highlights the need for crop-specific optimization. Key considerations include: (i) Zn accumulation in edible plant parts; (ii) impacts on soil microbial communities; (iii) leaching into groundwater; and (iv) effects on non-target organisms. Our study did not address these factors, representing important limitations. Future research should include long-term soil column studies, microbial community profiling, and bioaccumulation assessments.

## Conclusion

This study demonstrates the synthesis of zinc oxide nanoparticles using an endophytic *Aspergillus niger* isolate from *Celastrus paniculatus*. Comprehensive characterization confirmed the formation of crystalline, pure-phase ZnO NPs with a biomolecular corona contributing to stability. The NPs exhibited multiple in vitro bioactivities: antioxidant activity (IC₅₀ = 42.7 µg/mL), antibacterial effects (MIC = 0.41–3.33 µg/mL against tested strains), antifungal activity (EC₅₀ = 48.3 µg/mL against *F. oxysporum*), and modest plant growth promotion in rice seedlings at 2 µM concentration.

However, several important limitations must be acknowledged. The antimicrobial activity, while notable, does not exceed that of conventional antibiotics when benchmarked appropriately. The plant growth effects, though statistically significant, were modest in absolute magnitude. Crucially, the absence of mammalian cytotoxicity data and environmental fate studies precludes any definitive claims about biomedical or agricultural applications. The calcination step used in synthesis may have partially altered the biomolecular corona, and the mechanisms underlying observed bioactivities were not experimentally validated.

Therefore, while this work contributes a foundational characterization of this particular endophyte-nanoparticle system, the findings should be considered proof-of-concept rather than demonstration of practical applicability. Substantial additional research including cytotoxicity assessment, mechanistic studies, environmental fate analysis, and field trials will be necessary before any practical applications can be responsibly considered. The study nonetheless highlights the potential value of exploring endophytic fungi from medicinal plants as resources for green nanoparticle synthesis and provides a basis for future investigations.

**CRediT authorship contribution statement**.

**S.D.**: Writing-Original Draft, Validation, Software, Funding acquisition. **M.M.B.**: Methodology, Formal analysis, Data curation, Conceptualization. **U.M.A.**: Supervision, Resources, Conceptualization, Methodology, Software, Writing–Review & Editing. **A.T.**: Formal analysis, Writing - Review & Editing, Validation. All authors reviewed and approved the final manuscript.

## Supplementary Information

Below is the link to the electronic supplementary material.

Supplementary Material 1

## Data Availability

The data supporting this study are available from the corresponding author upon reasonable request. The fungal ITS sequence is deposited in GenBank under accession number KJ432863.1.
