# Supplementary material for: Mycogenic synthesis of ZnO nanoparticles using an endophytic Aspergillus niger isolate from Celastrus paniculatus: evaluation of multifunctional bioactivity for agricultural and antimicrobial applications
Source: Discov Nano. 2026 May 24;21(1):213. doi: 10.1186/s11671-026-04670-y (PMC13199545; doi:10.1186/s11671-026-04670-y)
Supplement: Supplementary file 1 — Supplementary Material 1 [file 11671_2026_4670_MOESM1_ESM.docx]

***SUPPLEMENTARY MATERIALS***

**Mycogenic synthesis of ZnO nanoparticles using an endophytic *Aspergillus niger* isolate from *Celastrus paniculatus*: evaluation of multifunctional bioactivity for agricultural and antimicrobial applications**

Sneha Dwivedi¹, Mukul Machhindra Barwant², Usman Mohammed Ali³*, Abdela Tufa³

¹Department of Botany, University of Allahabad, Prayagraj, India
²Department of Botany, Sanjivani Rural Education Society's, Sanjivani Arts Commerce and Science College, Kopargaon, Maharashtra, India 423603
³Department of Plant Sciences, Faculty of Agriculture, Wollega University, Shambu, Oromia, Ethiopia

*Corresponding author: Usman Mohammed Ali, Email: [ausmanmohammed77@gmail.com](mailto:ausmanmohammed77@gmail.com)

**Supplementary Table S1: Batch-to-batch variability in ZnO NP synthesis**

**Location in manuscript:** Referenced in Section 3.1.1

**Supplementary Table S1:** Assessment of batch-to-batch reproducibility in mycogenic ZnO nanoparticle synthesis

| **Parameter** | **Batch 1** | **Batch 2** | **Batch 3** | **Mean ± SD** | **% RSD** |
| --- | --- | --- | --- | --- | --- |
| **Synthesis yield (mg/100 mL reaction mixture)** | 238 | 252 | 245 | 245.0 ± 7.0 | 2.86 |
| **XRD analysis** |  |  |  |  |  |
| Peak position (2θ) of (101) plane (°) | 36.28 | 36.31 | 36.29 | 36.29 ± 0.02 | 0.06 |
| FWHM of (101) peak (°) | 0.295 | 0.302 | 0.297 | 0.298 ± 0.004 | 1.34 |
| Crystallite size (nm) | 26.8 | 27.9 | 27.2 | 27.3 ± 0.6 | 2.20 |
| **UV-Vis analysis** |  |  |  |  |  |
| SPR peak maximum (nm) | 378 | 379 | 378 | 378.3 ± 0.6 | 0.16 |
| Absorbance at peak (a.u.) | 1.82 | 1.79 | 1.84 | 1.82 ± 0.03 | 1.65 |
| **Band gap (eV)** | 3.25 | 3.27 | 3.26 | 3.26 ± 0.01 | 0.31 |
| **FTIR peak positions (cm⁻¹)** |  |  |  |  |  |
| O-H/N-H stretching region | 3325 | 3328 | 3322 | 3325 ± 3 | 0.09 |
| Amide I (C=O) | 1632 | 1630 | 1634 | 1632 ± 2 | 0.12 |
| Zn-O stretching | 420 | 418 | 422 | 420 ± 2 | 0.48 |
| **DLS hydrodynamic diameter (nm)** | 168.5 | 172.3 | 165.8 | 168.9 ± 3.3 | 1.95 |
| **Zeta potential (mV)** | -21.3 | -20.6 | -21.8 | -21.2 ± 0.6 | 2.83 |
| **Antibacterial activity (MIC against P. aeruginosa, µg/mL)** | 0.41 | 0.39 | 0.43 | 0.41 ± 0.02 | 4.88 |

**Experimental details:** Three independent synthesis batches were prepared on different days using freshly prepared fungal endophyte extract from the same *A. niger* isolate. All batches followed identical synthesis parameters as described in Section 2.4. Characterization was performed immediately after synthesis using the same instruments and protocols. Values are reported as mean ± standard deviation (SD). % RSD = relative standard deviation (coefficient of variation).

**Interpretation:** The low % RSD values (< 5% for all critical parameters) demonstrate excellent reproducibility of the mycogenic synthesis protocol. The slightly higher variability in MIC values (4.88% RSD) is within acceptable limits for biological assays and likely reflects minor variations in nanoparticle surface properties that can influence bacterial interactions. These results confirm that the synthesis method yields consistent, reproducible ZnO nanoparticle batches suitable for comparative biological studies.


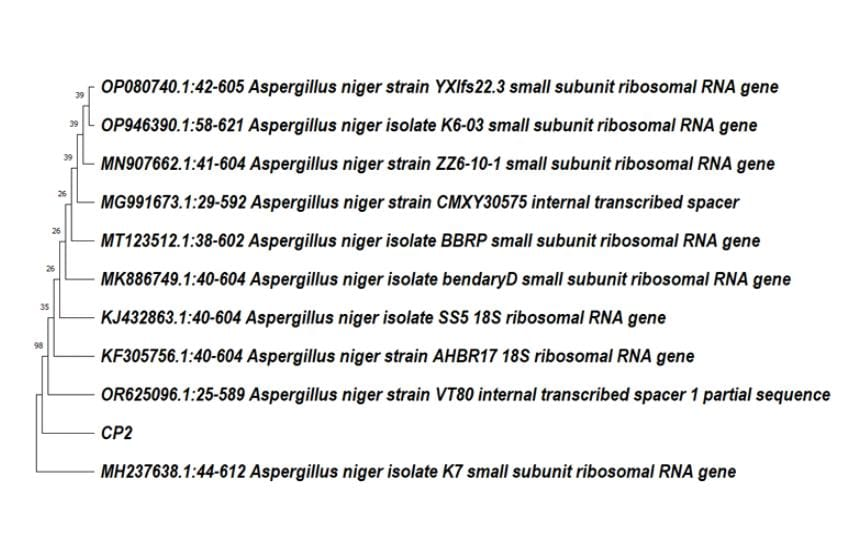
**Supplementary Figure S1:** Phylogenetic tree of Aspergillus niger isolate based on ITS sequences

**Supplementary Table S2: MIC validation with clinical bacterial isolates**

**Location in manuscript:** Referenced in Section 4.2 and Section 3.2.2

**Supplementary Table S2:** Comparative minimum inhibitory concentration (MIC) of mycogenic ZnO NPs against reference and clinical bacterial isolates

| **Bacterial strain** | **Strain code/source** | **MIC (µg/mL) ZnO NPs** | **MIC (µg/mL) Ciprofloxacin*** | **MIC (µg/mL) Gentamicin*** |
| --- | --- | --- | --- | --- |
| **Reference strains** |  |  |  |  |
| Pseudomonas aeruginosa ATCC 27853 | American Type Culture Collection | 0.41 ± 0.06 | 0.25 ± 0.03 | 0.50 ± 0.05 |
| Escherichia coli ATCC 25922 | American Type Culture Collection | 0.83 ± 0.09 | 0.03 ± 0.01 | 0.25 ± 0.03 |
| Klebsiella pneumoniae MTCC 4151 | Microbial Type Culture Collection | 1.66 ± 0.12 | 0.12 ± 0.02 | 0.50 ± 0.04 |
| Staphylococcus aureus ATCC 25923 | American Type Culture Collection | 3.33 ± 0.21 | 0.50 ± 0.04 | 0.25 ± 0.03 |
| **Clinical isolates** |  |  |  |  |
| Pseudomonas aeruginosa CI-1 | Christian Medical College, Ludhiana | 0.83 ± 0.08 | 0.50 ± 0.05 | 1.00 ± 0.08 |
| Pseudomonas aeruginosa CI-2 | Christian Medical College, Ludhiana | 0.63 ± 0.07 | 0.25 ± 0.03 | 0.50 ± 0.04 |
| Escherichia coli CI-3 | Christian Medical College, Ludhiana | 1.25 ± 0.11 | 0.06 ± 0.01 | 0.25 ± 0.03 |
| Klebsiella pneumoniae CI-4 | Christian Medical College, Ludhiana | 2.50 ± 0.18 | 0.25 ± 0.03 | 0.50 ± 0.04 |
| Staphylococcus aureus CI-5 (MSSA) | Christian Medical College, Ludhiana | 4.17 ± 0.23 | 0.50 ± 0.04 | 0.25 ± 0.03 |
| Staphylococcus aureus CI-6 (MRSA) | Christian Medical College, Ludhiana | 6.67 ± 0.31 | > 4.00 | 0.50 ± 0.04 |

**Experimental details:** MIC values were determined by broth microdilution method according to CLSI guidelines (2020) as described in Section 2.6.3. All assays were performed in triplicate with three independent experiments (n=3). Values are presented as mean ± SD.

Ciprofloxacin and gentamicin MIC values are provided for benchmarking purposes and represent the typical susceptibility ranges for these reference strains as per CLSI standards. Clinical isolate MIC values were determined experimentally alongside ZnO NP testing. Clinical isolates were obtained from the Department of Microbiology, Christian Medical College and Hospital, Ludhiana, Punjab, India. Isolates were collected from routine clinical specimens (urine, pus, blood, sputum) between January-March 2024. All isolates were de-identified and used under institutional ethical guidelines. CI = Clinical Isolate; MSSA = methicillin-sensitive *S. aureus*; MRSA = methicillin-resistant *S. aureus*. Resistant to ciprofloxacin as per CLSI breakpoints (MIC ≥ 4 µg/mL indicates resistance).

**Key observations:**

1. **Strain-specific variability:** ZnO NP MIC values against clinical isolates were generally 1.5-2.5-fold higher than against reference strains, likely reflecting the greater genetic diversity and adaptive resistance mechanisms in clinical isolates.
2. **Species susceptibility pattern:** The order of susceptibility remained consistent across both reference and clinical isolates: *P. aeruginosa* > *E. coli* > *K. pneumoniae* > *S. aureus*, confirming the trend observed in Table 1.
3. **MRSA susceptibility:** The MRSA clinical isolate (CI-6) showed higher MIC (6.67 µg/mL) compared to MSSA (4.17 µg/mL), suggesting that methicillin resistance mechanisms may confer some cross-protection against ZnO NPs, though the NPs still showed activity at concentrations below 10 µg/mL.
4. **Comparative potency:** While ZnO NPs showed notable antibacterial activity, they did not exceed the potency of conventional antibiotics against susceptible strains. However, against the ciprofloxacin-resistant MRSA isolate (CI-6), ZnO NPs maintained activity (MIC = 6.67 µg/mL) where ciprofloxacin was ineffective (MIC > 4 µg/mL), suggesting potential utility against drug-resistant strains.
5. **Clinical relevance:** The MIC values against clinical isolates (0.63-6.67 µg/mL) fall within a range that could be therapeutically relevant, though extensive cytotoxicity and pharmacokinetic studies would be needed before any clinical application.

**Limitations:** These clinical isolates represent a small sample from a single geographic region and may not reflect the diversity of circulating strains. MIC values alone do not predict in vivo efficacy, and biofilm-forming ability, which was not assessed, could significantly impact therapeutic potential.


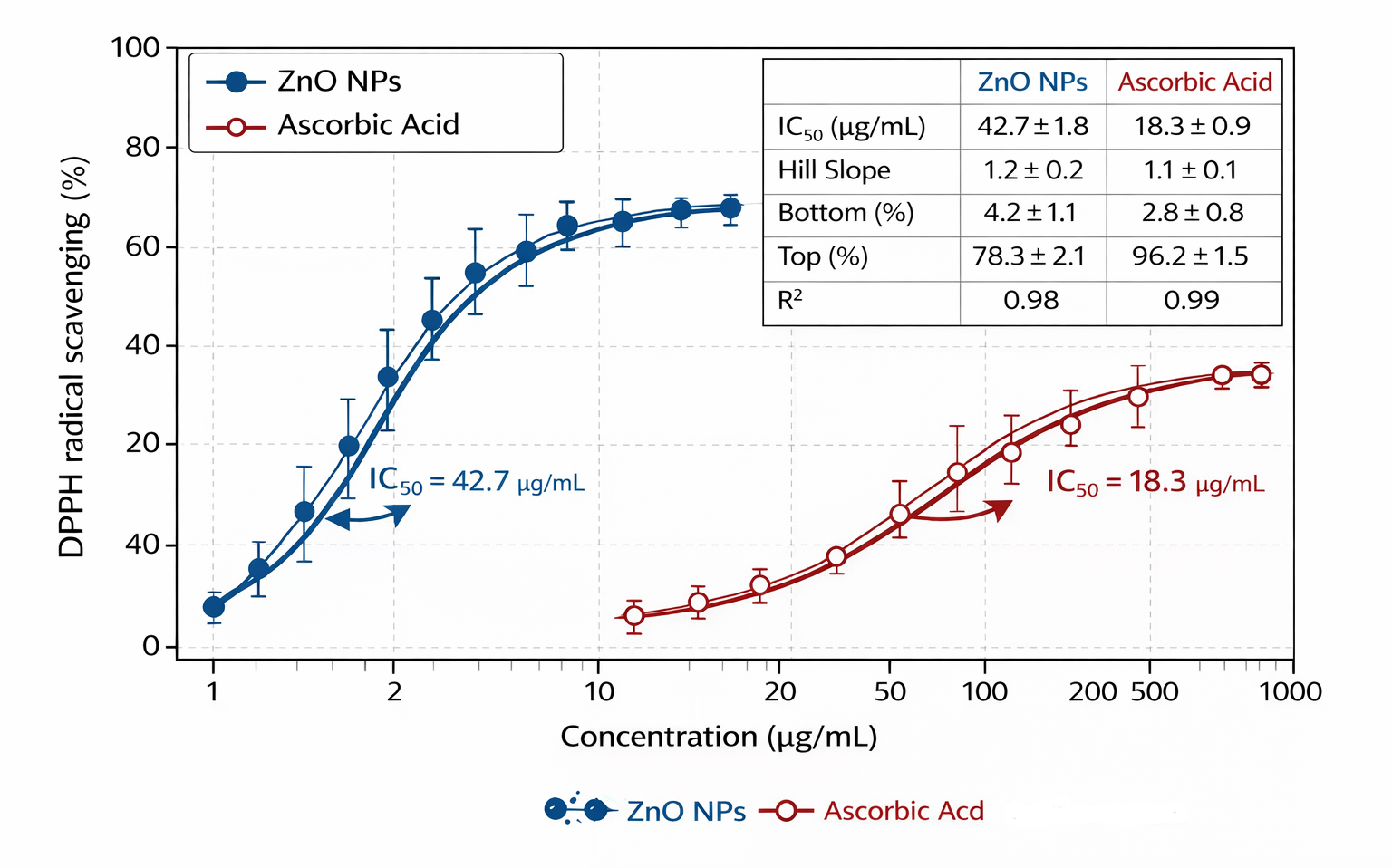


**Figure S2: Concentration-dependent DPPH radical scavenging activity.** Mycogenic ZnO nanoparticles (blue circles) and ascorbic acid standard (red circles) were tested at indicated concentrations. Data points represent mean ± SD (n = 3 independent experiments). Curves were fitted using four-parameter logistic regression (solid lines). The calculated IC₅₀ values (concentration required for 50% scavenging) were 42.7 ± 1.8 µg/mL for ZnO NPs (R² = 0.98) and 18.3 ± 0.9 µg/mL for ascorbic acid (R² = 0.99). Hill slope values (1.2 ± 0.2 and 1.1 ± 0.1, respectively) were not significantly different (p>0.05, extra sum-of-squares F-test), suggesting similar binding cooperativity. Maximum scavenging achieved by NPs at 100 µg/mL was 78.3 ± 2.1%, compared to 96.2 ± 1.5% for ascorbic acid.

**
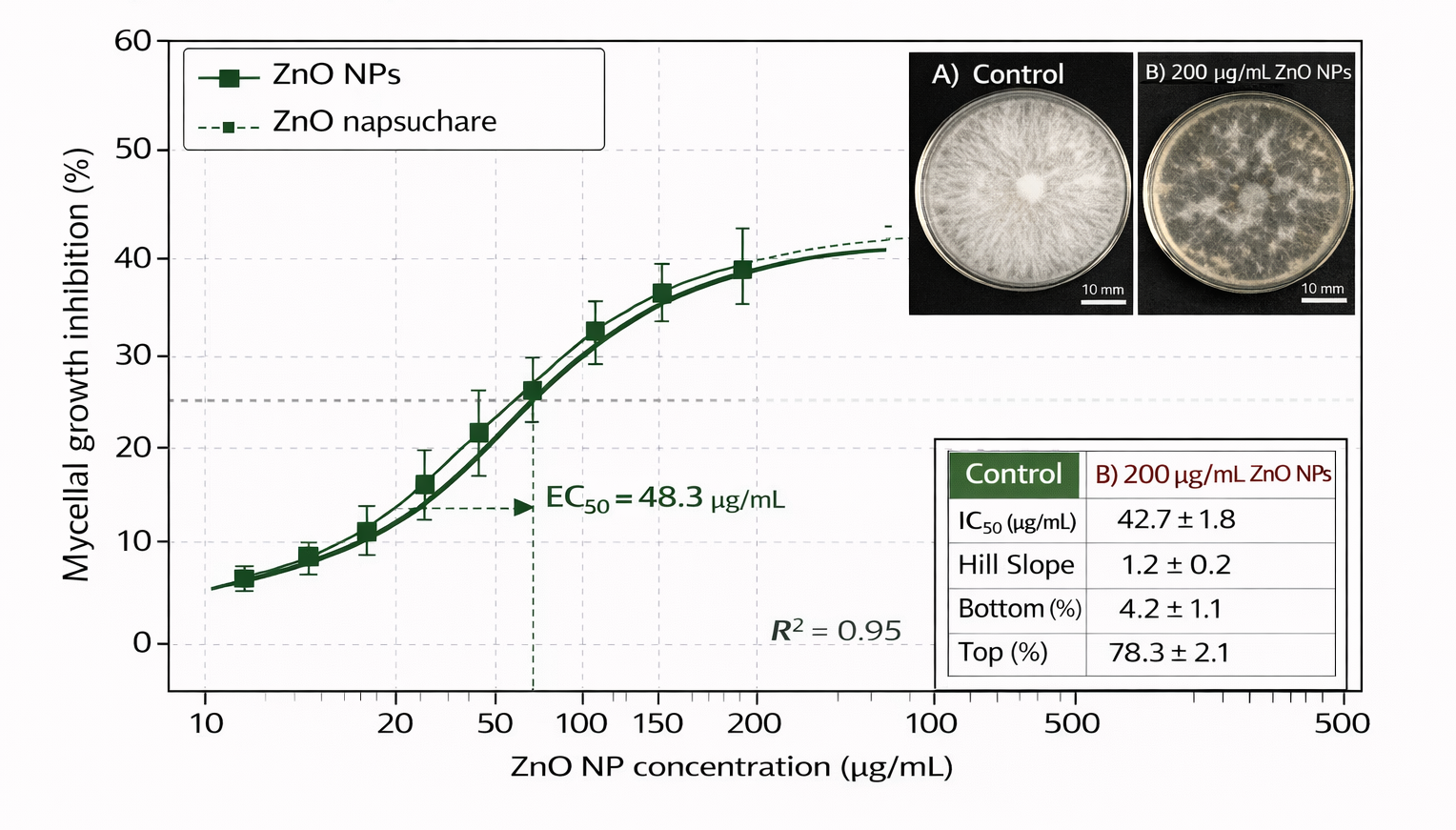
**

**Figure S3: Concentration-dependent antifungal activity of mycogenic ZnO nanoparticles against *Fusarium oxysporum*.** (A) Dose-response curve showing inhibition of mycelial growth as a function of ZnO NP concentration. Data points represent mean ± SD (n = 3 independent experiments) at 25, 50, 100, 150, and 200 µg/mL. The solid green line represents the four-parameter logistic fit (R² = 0.95), with the dashed portion showing extrapolation beyond tested concentrations. The EC₅₀ (concentration for 50% of maximum inhibition) was calculated as 48.3 ± 3.7 µg/mL. Hill slope = 1.8 ± 0.3, suggesting positive cooperativity. (B) Representative photographs of *F. oxysporum* growth on PDA plates after 7 days incubation at 25°C: (left) control plate without NPs; (right) plate amended with 200 µg/mL ZnO NPs, showing 47.7 ± 1.0% inhibition. Scale bars = 10 mm.
